# CSF Cholinergic Index, a New Biomeasure of Treatment Effect in Patients With Alzheimer’s Disease

**DOI:** 10.3389/fnmol.2019.00239

**Published:** 2019-10-11

**Authors:** Azadeh Karami, Maria Eriksdotter, Ahmadul Kadir, Ove Almkvist, Agneta Nordberg, Taher Darreh-Shori

**Affiliations:** ^1^Center for Alzheimer Research, Department of Neurobiology, Care Sciences and Society, Division of Clinical Geriatrics, Karolinska Institutet, Solna, Sweden; ^2^Karolinska University Hospital Huddinge, Theme Aging, Stockholm, Sweden

**Keywords:** Alzheimer’s disease, choline acetyltransferase, acetylcholinesterase, positron emission tomography (PET), cerebrospinal fluid (CSF), red blood cells (RBCs), nicotinic binding sites, galantamine

## Abstract

Alzheimer’s disease (AD) is a progressive disease with early degeneration of the central cholinergic neurons. Currently, three of four AD drugs act by inhibiting the acetylcholine (ACh) degrading enzyme, acetylcholinesterase (AChE). Efficacy of these drugs depends on available amount of ACh, which is biosynthesized by choline acetyltransferase (ChAT). We investigated whether treatment with a cholinesterase-inhibitor, galantamine, alters the relative levels of AChE to ChAT in cerebrospinal fluid (CSF) and whether levels of these CSF biomarkers correlate with *in vivo* AChE activity and nicotinic binding sites in the brain assessed by positron emission tomography (PET). Protein concentrations and activities of ChAT and AChE were measured in CSF of 18 patients with mild AD prior to and after 3 months of treatment with galantamine (*n* = 12) or placebo (*n* = 6), followed by nine additional months of galantamine treatment in all patients. A Cholinergic index was defined as the ratio of ChAT to AChE in CSF and was evaluated in relation to the *in vivo* AChE activity, the nicotinic binding sites and different measures of cognition. Besides an expected inhibition of AChE activity, galantamine treatment was accompanied by a mild increase in CSF ChAT activity. Thereby, the Cholinergic index was significantly increased in the *Galantamine group* (60% ± 14) after 3 months compared to baseline (*p* < 0.0023) or (*p* < 0.0004). This index remained high in *the Galantamine group* compared to baseline (54% ± 11) at 12 months follow-up, while it showed an increase in *the Placebo group* when they switched to active galantamine treatment (44% ± 14 vs. baseline, 61% ± 14 vs. 3 months, all *p*-values < 0.05). Furthermore, the *in vivo* brain AChE activity (assessed by PET) correlated with the CSF Cholinergic index at 12 months (*r* = 0.98, *p* < 0.001). The CSF Cholinergic index also correlated with ADAS-Cog and some other neuropsychological tests at 12 months. This is the first study assessing a CSF Cholinergic index in relation to treatment with a cholinesterase inhibitor. The treatment-specific increase in CSF ChAT activity suggests that cholinesterase-inhibitors may also increase the ACh-biosynthesis capacity in the patients. Additional studies are warranted to evaluate the utility of the CSF Cholinergic index as a biomeasure of therapeutic effect in AD.

## Introduction

The central cholinergic pathways have prominent roles in learning and memory. The severity of cholinergic deficits in patients with Alzheimer’s disease (AD) correlates with cognitive impairment, which have led to the development of cholinesterase inhibitors (ChEIs). The currently registered ChEIs as AD therapeutics putatively act by inhibiting the degradation of acetylcholine (ACh) in the synaptic cleft by the synaptic acetylcholinesterase (AChE), prolonging ACh’s action on its receptors (AChRs; Darreh-Shori and Soininen, 2010).

ChEIs are in use as symptomatic therapy in patients with AD for decades now. Still there are major gaps in our understanding of the *in vivo* biodynamic processes that may account for their limited clinical efficacies, possible disease-modifying effect or development of tolerance against these drugs.

There are three registered ChEIs for symptomatic treatment of dementia, namely donepezil (Winblad et al., 2001; Whitehead et al., 2004), rivastigmine (Rösler et al., 1999) and galantamine (Raskind et al., 2000, 2004; Tariot et al., 2000; Wilcock et al., 2003; Aronson et al., 2009), which have shown both short- and long-term benefits with regard to behavioral and cognitive measurements. The pharmacological properties differ among these ChEIs (Darreh-Shori and Soininen, 2010). Donepezil and galantamine are selective and rapidly reversible inhibitors of AChE, while rivastigmine is a pseudo-irreversible inhibitor of both AChE and its closely related enzyme, butyrylcholinesterase (BChE; Darreh-Shori and Soininen, 2010).

There are large amounts of AChE and BChE activities in blood and plasma. A hematopoietic splice variant of AChE exists in blood, anchored mainly on the red blood cells (RBC AChE) by a glycophosphatidylinositol anchoring protein. Measurement of changes in the RBC AChE activity following treatment with ChEIs has also been used to define the inhibition level of AChE in patients treated with ChEIs (Sramek and Cutler, 2000).

Several reports exist concerning the changes in the AChE and BChE activities and protein levels in blood and cerebrospinal fluid (CSF) following treatment with ChEIs (Nordberg and Svensson, 1998; Darreh-Shori et al., 2006, 2008; Nordberg et al., 2009). In CSF there are two splice variants of AChE, the synaptic (AChE-S) and the stress-associated read-through (AChE-R) variants (Darreh-Shori et al., 2004). Treatment with donepezil or galantamine causes a significant increase in the protein level of AChE-S in CSF of patients (Darreh-Shori et al., 2006, 2008; Nordberg et al., 2009). Through an integration of the changes in the CSF AChE-S protein relative to the corresponding changes in its activity in CSF (Darreh-Shori et al., 2006), we have shown that the CSF increase in the protein expression of AChE reflects at least partially the extent of *in vivo* AChE inhibition in the brain, assessed by positron emission tomography (PET; Darreh-Shori et al., 2008).

Nonetheless, such an increase in the AChE expression may also be a sign of development of a drug tolerance or is signifying a remodeling of the central cholinergic network in response to the prolonged action of ACh at the cholinergic synapses. Indeed, this is a highly plausible disease-modifying outcome since the primary target of the ChEIs is the membrane-anchored synaptic splice variant of AChE protein at the remaining functional cholinergic synapses (Lane and Darreh-Shori, 2015).

To investigate whether the observed increase of AChE protein expression in CSF of patients treated with ChEIs is a sign of drug tolerance or remodeling of the central cholinergic network, it is crucial to simultaneously investigate the changes in the level of the ACh-synthesizing enzyme, choline acetyltransferase (ChAT). This is because a remodeling and/or regeneration of the cholinergic synapses will most likely require extra expression of both ChAT and AChE. In this context, recent reports of the presence of a soluble variant of ChAT in both plasma and CSF has provided a unique opportunity (Vijayaraghavan et al., 2013 Karami et al., 2015) to monitor changes in soluble ChAT for assessing possible improvement of cholinergic neurons in the brain during ChEIs therapy.

In the current study, we hence investigated the changes in AChE and ChAT activities following treatment of a group of patients treated with placebo or galantamine for 3 months followed with galantamine treatment for all in 9–12 months. These changes were also analyzed in relation to available *in vivo* brain PET data, namely the number of nicotinic binding sites and AChE inhibition in different brain regions of the AD patients for up to 1 year.

These objectives are clinically important by providing information about short- and long-term efficacy as well as the underlying mechanism of development of tolerance or delaying the progression of AD during long-term treatment with ChEIs.

## Materials and Methods

### Study Design and Patients

**Figure 1:**
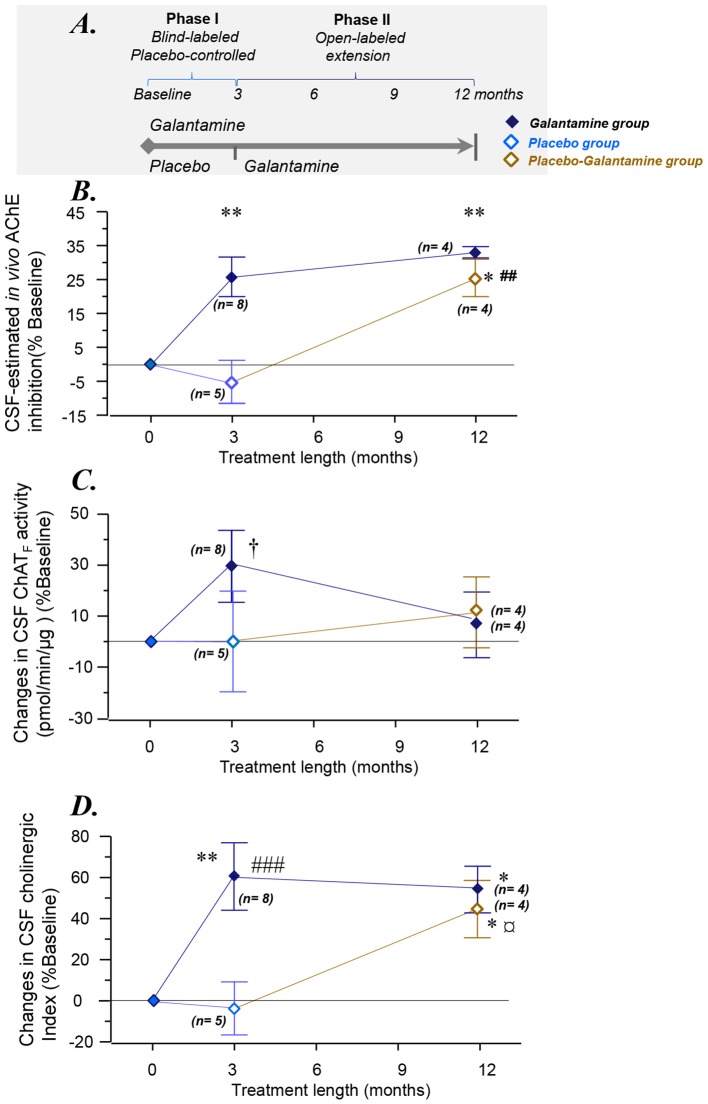
Changes in the overall functional ChAT and AChE activities in CSF. **(A)** Schematic illustration of the galantamine study design (Kadir et al., 2008). **(B)** Changes in the total AChE activity in the CSF. **(C)** Changes in CSF ChAT_F_ activity. **(D)** Overall changes as a Cholinergic index in CSF after 3–12 months of the study. *p < 0.05, and **p < 0.01 signify differences compared to baseline. ^##^*p* < 0.01 and ^###^*p* < 0.001 signify differences compared to *the Placebo group* at 3 months. ^†^*p* < 0.099 compared to baseline or *the Placebo group*. ^¤^*p* < 0.08 compared to *the Placebo group* (when *n* = 5, see also Supplementary Figures S1A,B). AChE = acetylcholinesterase. ChAT = choline acetyltransferase. CSF ChAT_F_ activity = the overall functional ChAT activity expressed in pmol/min/μg ChAT protein in the samples. CSF AChE_F_ activity = the overall functional AChE activity expressed in nmol/min/μg AChE protein in the samples. CSF Cholinergic index = ratio of ChAT_F_ activity to AChE_F_ in CSF.

Details of the study have been described elsewhere (Darreh-Shori et al., 2008; Kadir et al., 2008). Briefly, 18 mildly demented AD patients [mean age 69 ± 2 years, Mini Mental State Examination (MMSE) 26 ± 1, duration of disease 4 ± 1 years] were included. A schematic presentation of the study design is shown in Figure 1A. The first 3 months of the study was a double-blind placebo-controlled study where six AD patients received placebo and 12 patients received galantamine in flexible doses (16–24 mg daily), followed by 9 months treatment with galantamine for all patients. The patients were recruited at the Danderyd Hospital, and the memory clinic of Karolinska University Hospital Huddinge, Stockholm, Sweden. The diagnosis of AD was made in accordance with the National Institute of Neurological and Communication Disorders and Stroke-AD and Related Disorders Association (NINCDS-ADRDA) criteria (McKhann et al., 1984). Neuropsychological tests were performed throughout the study, at baseline, and after 3 and 12 months follow-up. Blood and CSF samples were obtained at baseline, at 3 and at 12 months.

All patients and their caregivers provided written informed consent to participate in the study, which was conducted according to the Declaration of Helsinki and subsequent revisions. The Ethics Committee of Huddinge University Hospital, Sweden approved the study.

### Collection of Blood and CSF Samples

To evaluate cholinesterase activities in RBC and CSF, whole blood and CSF samples were collected from all patients at baseline (pre-drug samples). Whole-blood was collected in standard tubes containing EDTA as anticoagulant. Then, blood was also collected after 3 and 12 months during the study. CSF samples, taken at baseline and after 3 and 12 months of treatment, were collected by lumbar puncture in the L3/L4 or L4/L5 interspace at mornings between 9–12 a.m. All CSF samples were kept frozen in small aliquots at –80°C, until the assay. More detailed information have been given previously (Darreh-Shori et al., 2008; Kadir et al., 2008).

### Measurement of CSF ChAT Activity and Protein Concentration

CSF ChAT activity was measured by a colorimetric assay (Vijayaraghavan et al., 2013) in the baseline samples as well as in samples collected after 3 and 12 months follow-up. A modified version of the assay protocol was used, which sequentially quantifies both the activity and amount of ChAT protein, as well as the endogenous choline concentration in biological samples, described in detail below.

#### ChAT Assay Preparations

The wells of high binding 384-well microtiter plates (Nunc Maxisorb, Denmark) were coated overnight at 4°C with 75 μl of monoclonal anti-human ChAT antibody (MAB3447, R&D System, UK; at 1:250 dilution in carbonate buffer, pH 9.8). The wells for choline standards and their blanks were not coated with the capturing antibody. On the following day, the plates were washed with PBS and tapped dry for the next step (as described below in the assay procedure).

Undiluted CSF samples (10 μl/well) were applied both unmodified (i.e., in native conditions, in triplicates) and as modified denatured samples (also in triplicate). The denatured samples served as controls for both the ChAT activity and the endogenous concentration of choline in the samples. The denatured CSF samples were prepared by heating in a thermal cycler, with three cycles of 8 min at 98°C, and 30 s at 60°C.

Two series of standards were used on each plate, a choline standard sets for quantifying the choline concentration in the wells, and a ChAT protein standard series for quantifying the amount of ChAT protein in the samples. The choline standards were prepared by two-fold serial dilutions of choline chloride solution in TBS buffer (10 mM Tris-HCl, 0.9% sodium chloride, 1 mM EDTA and 0.05% Triton X-100, pH 7.4). The highest choline standard point was 50 μM, and 50 μl/well (in triplicates) of each choline standards were applied into the wells of the microtiter plate. As the ChAT protein standard, a calibrated pooled human plasma sample was used. Like the CSF samples, the ChAT protein standards were applied both as native (unmodified) and as denatured samples (10 μl/well, all in triplicates). The highest ChAT protein standard had a concentration of 1.14 μg/ml, which was prepared by 50 times dilution of the calibrated pooled plasma sample. The denatured standards were prepared as described above for CSF samples.

The ChAT assay requires two master-mix cocktails: ***Cocktail-A*** and ***-B***. The ***Cocktail-A*** was prepared in the TBS buffer, and contained 18.75 μM of choline chloride (C1879, Sigma-Aldrich), 0.75 mM of eserine hemisulfate (E8625, Sigma-Aldrich), 1.25U/mL of phosphotransacetylase (P2783, Sigma-Aldrich), 8.75 mM of acetyl phosphate (P2783, Sigma-Aldrich), and 62.5 μM acetyl-coenzyme A (A2181, Sigma-Aldrich). The Cocktail A was incubated for 10–15 min at 38°C prior to the assay procedure. The ***Cocktail-B*** was prepared in PBS (50 mM phosphate buffered saline, pH 7.4), and contained 0.93 U/ml of choline oxidase (from a 50 U/ml stock, C5896, Sigma-Aldrich), 1/5,000 Streptavidin horseradish peroxidase (P6782 Sigma-Aldrich), 6.3 mM of phenol (P3653, Sigma-Aldrich), and 3 mM of 4-aminoantipyrine (A4382, Sigma-Aldrich). Both cocktails should be prepared freshly.

#### ChAT Assay Procedure

The unmodified and denatured CSF samples (10 μl/well), the ChAT protein standards (10 μl/well), or the TBS buffer (10 μl/well, as choline-controls) were applied to the wells of the anti-ChAT antibody pre-coated microtiter plate. The choline standards (50 μl/well) were applied to un-coated wells on the plate. Then, 40 μl/well of the Cocktail-A was applied to all wells but the choline standards, using a multichannel pipette. The plate was then sealed and incubated for 2 h in a humid chamber at 38.5°C under gentle shaking. The plate was cooled briefly on ice-water bath and centrifuged gently. The sealing tape was removed and 25 μl/well of the ***Cocktail-B*** was added to all wells, including the wells containing the choline standards. The reaction was then monitored continuously at 1–2 min intervals at 500 nm for changes in absorbance for as long as was deemed necessary (usually 15–30 min) using a Tecan Infinite M1000 microplate reader. This step quantifies both the ChAT activity and the choline concentration in the samples.

After this step, the plate was sealed and incubated overnight at 4°C. On the following day, the plate was washed three times for 5 min with TBS-T^0.05%^ (10 mM Tris-HCl, pH 7.4, 0.9% sodium chloride, 0.05% Tween 20), and incubated with 100 μl/well of a blocking buffer (TBS-T^0.05%^ containing 5% BSA and 0.01% sodium azide) for 30 min at 38°C in a humid chamber under gentle shaking. The plate was then washed as before, and incubated for 60 min with 50 μl/well of a working dilution of 1:1,700 of the detecting anti-ChAT antibody (rabbit anti-ChAT polyclonal antibody, PAB 14536, Abnova, Taiwan, prepared in TBS-T^0.05%^, containing 1% BSA and 0.01% sodium azide) at 38°C in a humid chamber under gentle shaking. Then 25 μl/well of a working solution of a secondary swine anti-rabbit polyclonal antibody (1:2,000, D0306, prepared in TBS-T^0.05%^, containing 1% BSA and 0.01% sodium azide) was added to the wells and incubated for two more hours at room temperature under gentle shaking. The plate was washed four times with TBS-T^0.05%^ and one time with 1 M diethanolamine buffer (pH 9.8, containing 1 M magnesium chloride and 0.01% sodium azide). Finally, 75 μl/well of a solution of the alkaline phosphatase substrate [1 mg/mL of p-nitrophenyl phosphate (73724, Sigma-Aldrich), in the diethanolamine buffer]. The absorbance was read at 405 nm until the absorbance of plasma standard reached 2–2.5.

ChAT activity (pmol/min/mL samples) was calculated according to the following formula: **ChAT activity = (C_d_ − C_n_)/*t*** * *V*_s_, in which *C*_d_ and *C*_n_ are the measured choline concentrations in the wells (in pmol) containing the denatured and native samples, respectively, *t* is the incubation time with the ***Cocktail-A*** (in minutes) at 38°C, and *V*_s_ is the 10 μl volume of the samples applied into the wells (in mL). The ChAT protein was determined as ng/mL CSF from the second part of the assay protocol, which is similar to a conventional ELISA assay.

The ChAT activity can also be expressed in relation to its protein concentration by dividing the ChAT activity (expressed in pmol/min/mL) to the ChAT protein concentration in the samples (ng/mL). For the simplicity, this parameter will be referred to in the following texts as **functional** **ChAT activity** (ChAT_F_). It should be noted that the ChAT_F_ activity has the unit of pmol/min/ng of ChAT protein. This integration of ChAT activity and protein is necessary for direct comparison between changes in ChAT activity and inhibition of AChE following galantamine (see below).

### Measurement of RBC and CSF AChE Activities

The methodological details for measuring RBC and CSF AChE activities have been described previously (Darreh-Shori et al., 2006, 2008).

The RBC AChE activity was measured at baseline, and after 3, 6, 9 and 12 months of treatment with galantamine. The CSF AChE activity was measured at baseline and after 3 and 12 months of follow-up.

### CSF-Estimation of AChE Inhibition

The total protein level of the AChE in CSF was measured using a functional-ELISA method previously described in detail for the AChE-R and AChE-S variants (Darreh-Shori et al., 2006, 2008).

The inhibition of the total AChE activity in the CSF of the AD patients was calculated by integrating the CSF AChE activity measured directly by the colorimetric assay with the amount of functionally intact AChE protein in CSF as previously described for the AChE variants’ activities (Darreh-Shori et al., 2006, 2008).

### *In vivo* Brain AChE Level by PET

PET was used with the tracer N-[^11^C] methyl-piperidin-4-yl propionate (^11^C-PMP), which is an ACh analog, to measure the reduction of cerebral cortical AChE activity in the brain. In the ^11^C-PMP-PET, the rate constant *k*_3_ for hydrolysis of ^11^C PMP by AChE according to a kinetic model developed in Kuhl et al. (1999). The methodological details are described in Kadir et al. (2007). The *in vivo* brain AChE activity was determined as *k*_3_ values (min^−1^) in different brain regions at the follow-up intervals. Thus, the *k*_3_ value has a direct relationship with AChE activity. For cortical regions, the AChE activity was calculated as the average of *k*_3_ values from the “region of interest” (ROI) in the anterior cingulate, frontal, frontal association, parietal, parieto-temporal, temporal, primary visual, sensory motor and temporal medial lobe cortices of the left and right brain hemispheres.

### *In vivo* Brain Level of Nicotinic Acetylcholine Receptors (nAChR) by PET

PET with ^11^C-nicotine tracer was used to measure the nicotinic binding sites *in vivo* in the brain. The details of ^11^C-nicotine PET assessment have been described previously (Kadir et al., 2008). Briefly, the number of nicotinic binding sites (*k*_2_*) was calculated in the brain regions using the binding of the tracer (S)(−) ^11^C-nicotine adjusted for regional cerebral blood flow (${\text{H}}_{2}^{15}\text{O}$). These measurements were performed at baseline, after 3 weeks, 3 and 12 months of follow-up. The relative changes in the nicotinic binding sites at the follow-ups compared to baseline was calculated using the following formula [%1-*k*_2_* = 100−(*k*_2_*_ (f)_/*k*_2_*_ (b)_ ×100), f and b indicate the *k*_2_* values at the follow-ups and the baseline, respectively]. The *k*_2_* has an inverse relationship with the number of nicotinic binding sites in the brain.

### Neuropsychological Assessments

To evaluate the effect of galantamine treatment on cognitive function, neuropsychological assessments were performed at baseline (pre-treatment), and after 3 and 12 months of treatment. The details are reported previously (Kadir et al., 2008). Briefly, global cognition was assessed using the MMSE, and the cognitive subscale of the AD Assessment Scale (ADAS-cog). A higher ADAS score indicates a greater degree of cognitive impairment. A battery of neuropsychological tests, addressing different cognitive domains (episodic memory, attention/executive ability, and visuospatial ability) were performed throughout the study. Episodic memory was evaluated using two measures from the Stockholm Gerontology Research Center test of memory for words: (1) the number of correct responses in free recall of words; and (2) the d-prime value (an integration of correct responses and false alarms following decision theory) in recognition of words. Attention domain was assessed using three measures: (1) the number of correct responses in the Digit Symbol response test from the revised Wechsler Adult Intelligence Scale (Wechsler, 1981); (2) the time needed to complete the Trailmaking A; and (3) the number of correct responses in the Trailmaking B test. These measures are known to assess abilities associated with frontal-subcortical brain activity (Cummings, 1993). Visuospatial ability domain was evaluated by recording the number of correct responses in reading and setting a clock. This test reflects parietal lobe function (Cahn-Weiner et al., 1999). To reduce the number of statistical analyses, these data were Z-transformed and the overall composite *Z*-scores of the three cognitive domains were calculated as described before (Darreh-Shori et al., 2011).

### Statistical Analysis

Data are expressed as mean values and standard error of the mean (SEM) or otherwise as stated. The effects of treatment at different time intervals compared to baseline were assessed using repeated measures (RM) analysis of variance (ANOVA) for the double-blind phase as well as open-label phase. The comparison between the groups was done using ANOVA analysis. A significant ANOVA result (*p* < 0.05) was followed by the Bonferroni–Dunn *post hoc* analysis that tested the significance of results at each time point compared with baseline or between groups. Correlation analyses were performed using correlation *Z*-tests. When deemed necessary, the correlations were asserted using the nonparametric Spearman Rank Correlation and were visualized using simple regression plots.

## Results

### The Study Design and Groups

The study design and the demographic of the patients are shown schematically in Figure 1A and Table 1, respectively. The Phase I of the study was 3 months during which the patients received either galantamine (*the Galantamine group*) or placebo (*the Placebo group*). During the Phase II of the study, the patients in *the Galantamine group* continued on galantamine for a total of 12 months. At the beginning of the phase II of the study, *the Placebo group*, however, started on receiving galantamine and continued on this drug for a total of 9 months (*the Placebo-Galantamine* group).

**Table 1:** Demographic data of the placebo and galantamine-treated groups at baseline.

	Placebo group (*n* = 6)	Galantamine group (*n* = 12)	Total (*n* = 18)
Gender (F/M)	3/3	5/7	8/10
Average age in	65.8 ± 3.7	70.9 ± 2.7	69.2 ± 2.2
years (range)
ApoE 4 carriers (+/−)	3/2	7/3	10/5
Duration of disease (years)	2.2 ± 0.7	5.1 ± 1.1	4.1 ± 0.8
Education, mean years (range)	12.8 ± 0.9	10.9 ± 1.1	11.4 ± 0.9
ADAS-cog	19.0 ± 1.1	28.0 ± 3.4	26.2 ± 0.7
MMSE	27.3 ± 0.8	25.6 ± 1.0	26.2 ± 0.7

*Values correspond to mean ± SEM. F, females and M, males. MMSE, Mini-Mental State Examination. ADAS-cog/13, Alzheimer’s Disease Assessment Scale-cognitive subscale. Although the placebo randomized patients appear younger, having shorter duration of the disease, higher MMSE scores and lower ADAS-cog scores at baseline compared with the galantamine group, no statistical significant difference was observed with regards to age, gender, level of education, duration of disease, MMSE and ADAS-cog scores at baseline (all *p* > 0.10 except for ADAS-cog, *p* = 0.09; Kadir et al., 2008)*.

### Patient Dropout

Of the initial 18 subjects (Table 1), during the double-blind phase, one subject (in *the Galantamine group*) was withdrawn as a result of second-degree atrioventricular block, considered possibly related to galantamine. Overall, 17 subjects completed the double blind Phase I of the study which was for 3 months. During the second phase, i.e., the open-label phase II, three more subjects were withdrawn: one from *the Placebo-Galantamine group* owing to chronic lymphocytic leukemia (an event considered unrelated to galantamine), and the others from the Galantamine arm of the study: one patient due to cardiac arrhythmia and syncope (considered possibly related to galantamine), and one patient as a result of non-compliance. Of the remaining 14 patients, one could not complete the neuropsychological tests at 12 month due to myocardial infarction (which relation to galantamine treatment was considered doubtful). It should be also noted that due to technical issues with PET scan validation and data acquisition, a valid AChE PET scan was available for only 15 patients (five in *the Placebo group* and 10 in *the Galantamine group*) at baseline and 3 months but 11 valid AChE PET scans at 12 months. In case of ^11^C-nicotine PET scan, data were for similar technical issues available for 17 patients (six in *the Placebo group* and 11 in *the Galantamine group*) at baseline and 3 months but 14 valid ^11^C-nicotine PET scans at 12 months. Thus, in principle there were a total of four dropout and 14 subjects completed the 12 months study.

Furthermore and particularly relevant to the CSF analyses, at the time of conducting the CSF ChAT measurements there were not enough CSF left either at the 3 months or the 12 months follow-up for some of the patients. Given that the statistical analyses were done on repeated measured analyses only the subjects that had data at baseline, 3 months and/or 12 months could be compared.

### Estimated RBC and CSF AChE Inhibition

The descriptive results regarding the changes in the RBC and CSF AChE activities are reported elsewhere (Darreh-Shori et al., 2008). Briefly, a weak to moderate inhibition of RBC AChE activity was observed in *the Galantamine group* at the 3-months follow-up (11 ± 1.4%, *n* = 11). No significant changes in the RBC AChE activity was observed in *the Placebo group* at 3 months. At the 12-month follow-up, however, the RBC AChE inhibition in *the Placebo-Galantamine group* was similar to the inhibition level in *the Galantamine group* (10 ± 4%, *n* = 6 vs. 10 ± 1.3%, *n* = 8, respectively).

The specific changes in the synaptic and read-through AChE splice variants proteins have been reported previously (Darreh-Shori et al., 2008). Here, we estimated the inhibition levels of total AChE activity in CSF by integrating the activity (in nmol/min/mL) and protein levels of AChE (ng/μl), as previously described (Darreh-Shori et al., 2014).

This estimated CSF AChE inhibition is shown in Figure 1B. The CSF AChE inhibition was 26 ± 6% and 33 ± 2% in *the Galantamine group* at 3 and 12 months of the study, respectively (all *p*-values < 0.003, Figure 1B). No inhibition of CSF AChE activity was observed in *the Placebo group* at 3 months (Figure 1B). In *the Placebo-Galantamine group*, the AChE activity was however inhibited in the CSF by 25 ± 6% at the 12 months follow-up when these patients had been on galantamine treatment for a total of 9 months (*p* < 0.02 compared to baseline, and *p* < 0.003 compared to the 3 months).

### Estimated Activity of the Acetylcholine-Synthesizing Enzyme, ChAT in CSF

#### Functional CSF ChAT Activity

Since the CSF AChE activity was normalized to the AChE protein levels, it was necessary to also integrate the overall ChAT activity (in pmol/min/mL CSF) with its protein in CSF (in ng/mL), to estimate changes in the level of functional ChAT activity (i.e., ChAT_F_) in CSF.

*At 3 months*, the ChAT_F_ activity showed about 30% numerical increase among *the Galantamine group* compared to baseline (*p* < 0.099, *n* = 8, Figure 1C), or *the Placebo group* (*n* = 5). Noteworthy, repeated-measure ANOVA includes only four subjects in *the Placebo group* who had 12-months data. These analyses indicated that *the Galantamine group* had 47% higher levels of ChAT than *the Placebo group* (*n* = 4, *p* < 0.05, Supplementary Figure S1). Considering that galantamine is a drug without any direct effect on ChAT_F_ activity, the observed increased in ChAT_F_ activity might indicate an expected effect of treatment in the stimulation of the cholinergic system.

*At 12 months* in *the Galantamine group*, the CSF ChAT_F_ activity did not differ compared to the baseline (Figure 1C). Among *the Placebo-Galantamine group* who were initially on placebo treatment, baseline, 3-month and 12-month CSF samples were available from four out of five of the patients. The Supplementary Figure S1A illustrates an accordingly modified version of Figure 1C. As it is easier to appreciate from the Supplementary Figure S1A, the CSF ChAT_F_ activity showed 29% significant increase in *the Placebo-Galantamine group* at the 12 months follow-up compared to their 3 months follow-up (*n* = 4, *p* < 0.02, Supplementary Figure S1A).

#### CSF Cholinergic Index

Cholinergic signaling is most likely regulated through a balance between synthesis, release and degradation of ACh. We hence defined a *CSF Cholinergic index* as a ratio by dividing the functional levels of ChAT (in pmol/min/μg) to that of AChE (in nmol/min/μg) in CSF (ChAT_F_/AChE_F_ activities). Thus, an increase in this ratio means a net increase in ACh levels.

*At 3 months*, the Cholinergic index differed significantly between *the Galantamine* and *the Placebo* groups (64 ± 14%, *p* < 0.0004).* The Galantamine group* showed also about 60% significant increase when compared to baseline (*p* < 0.003, Figure 1D). No significant change was observed in the CSF Cholinergic index in *the Placebo group* at 3 months compared to baseline (4 ± 13%, *p* < 0.3, Figure 1D).

*At 12 months* and in *the Galantamine group*, the Cholinergic index was higher compared to the baseline (54 ± 11%, *p* < 0.03), indicating that in this group the improved cholinergic index was maintained for up to 12 months of galantamine treatment. Most importantly, at the 12 months follow-up, a similar increase in this index was observed in *the Placebo-Galantamine group*. This increase was significant compared to both the baseline (44 ± 14%, *n* = 4, *p* < 0.05, Figure 1D) and compared to 3-month levels when these patients were still on placebo treatment (61 ± 14%, *n* = 4, *p* < 0.02, Figure 1D and its modified version Supplementary Figure S1B).

### Correlations Between the *in vivo* Brain AChE Activity (k_3_) by PET and the AChE and ChAT Activities in CSF

*At baseline*, there were no significant correlations between ChAT_F_ activity or the Cholinergic index in CSF and the *in vivo* brain AChE activity (assessed as *k*_3_) by PET.

*At 3 months*, the *in vivo* AChE activity (*k*_3_) in the whole brain correlated with the CSF ChAT_F_ activity (*r* = −0.6, *n* = 12, *p* < 0.05), and the CSF Cholinergic index (*r* = − 0.7, *n* = 12, *p* < 0.003), as assessed in all patients as one group.

**Figure 2:**
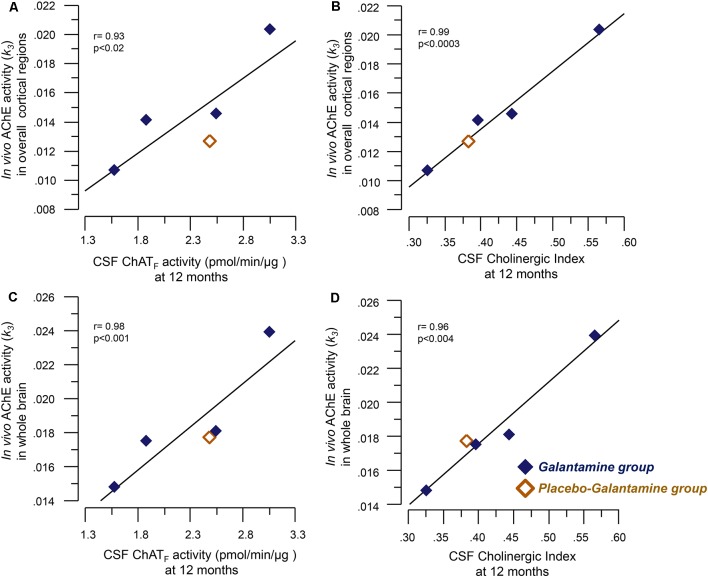
Correlations between the *in vivo* brain AChE activity (*k*_3_) by PET and the CSF cholinergic markers. Panel **(A)** illustrates correlations between the *in vivo* AChE activity (*k*_3_) overall cortical regions and the CSF ChAT_F_ activity at 12 months. Panel **(B)** shows the corresponding correlation with the Cholinergic index in CSF. Graphs C and D show correlations between the *in vivo* AChE activity (*k*_3_) in the whole brain and CSF ChAT_F_ activity **(C)** and the Cholinergic index **(D)** in CSF at 9–12 months galantamine treatment. The *k*_3_ value has a direct relationship with AChE activity, i.e., a high *k*_3_ value means a high *in vivo* AChE activity. CSF ChAT_F_ activity = the overall functional ChAT activity expressed in pmol/min/μg ChAT protein in the samples. CSF AChE_F_ activity = the overall functional AChE activity expressed in nmol/min/μg AChE protein in the samples. CSF Cholinergic index = ratio of ChAT_F_ activity to AChE_F_ in CSF.

*At 12 months*, significant correlations were observed between the *in vivo* AChE activity (*k*_3_) and the ChAT_F_ activity as well as the Cholinergic index in CSF, which are summarized in Table 2 and shown graphically in Figure 2.

**Table 2:** Correlations between the *in vivo* AChE activity (*k*_3_), cognitive measures, the CSF ChAT_F_ activity and the Cholinergic Index after 12 months.

		CSF ChAT_F_ activity		CSF Cholinergic index	
*in vivo* AChE activity in the brain (*k*_3_)		*r* (*n* = 5)	*p* < 0.05	*r* (*n* = 5)	*p* < 0.05
Cortical regions	*L*	0.92	**0.023**	0.98	**0.0009**
	*R*	0.90	**0.040**	0.95	**0.009**
Overall brain regions	*L*	0.92	**0.022**	0.96	**0.007**
	*R*	0.83	**0.090**	0.84	*0.084*
Average cortical regions		0.93	**0.020**	0.99	**0.0003**
Average overall brain regions		0.94	**0.015**	0.97	**0.004**
*Whole brain*		*0.96*	**0.0044**	*0.98*	**0.0012**
**Cognitive tests**		***r* (*n* = 8)**	***p* < 0.05**	***r* (*n* = 8)**	***p* < 0.05**

*MMSE Score*		*0.7*	**0.03**	*0.6*	*0.1*
*ADAS-Cog*		*−0.81*	**0.013**	*−0.83*	**0.008**
*Neuropsychology tests: Attention Domain*		*0.56*	*0.15*	*0.40*	*0.34*
* Memory Domain*		*0.50*	*0.22*	*0.77*	**0.022**
* Visuospatial Domain*		*0.85*	**0.0052**	*0.72*	**0.040**

*k_3_ = the binding constant calculated as described (Kadir et al., 2008). The *k*_3_ rate constants for the *in vivo* AChE activity are shown in Figure 2. All *r*-values are correlation coefficient. Some of the correlations are illustrated graphically in Figure 2 and Figure 6. PET, Positron emission tomography; CSF, Cerebrospinal fluid; R and L the right and left brain hemispheres. A large K3 means high *in vivo* AChE activity. CSF ChAT_F_ activity refers to choline acetyltransferase activity divided by its protein concentration; Cholinergic Index, refers to ratio of CSF ChAT_F_ activity/CSF AChE_F_ activity; MMSE Score, the mini-mental state exam; ADAS-Cog, Alzheimer’s Disease Assessment Scale*.

### Correlations Between the *in vivo* ^11^C-Nicotine PET in the Brain and Activities of AChE and ChAT in CSF

#### At Baseline Prior to Commencing Treatment

**Figure 3:**
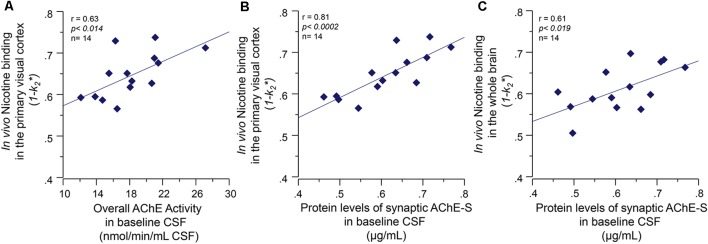
Baseline co-dependency of *in vivo* nicotinic binding sites in the brain and AChE levels in CSF. Positive correlation between the *in vivo* nicotinic binding sites in the primary visual cortex and AChE activity **(A)** and the protein levels of the synaptic AChE-S variant **(B)** in the CSF of the Alzheimer’s disease (AD) patients collected at baseline prior to the galantamine treatment. Panel **(C)** illustrates the positive correlation between the CSF AChE-S protein and the *in vivo* ^11^C-nicotine binding in *the whole-brain* measured by PET. Here, the values are given as *1−k*_2_ since *k*_2_* values have an inverse relationship with ^11^C-nicotine binding, i.e., a low *k*_2_* values mean a higher ^11^C-nicotine binding level. CSF, cerebrospinal fluid; PET, positron emission tomography; AChE-S, synaptic acetylcholinesterase splice variant proteins.

*At baseline*, the CSF AChE activity correlated positively with a number of nicotinic binding sites in the pons (*r* = 0.60, *p* < 0.03) and the left and right primary visual cortex (*r* = 0.63, *p* < 0.014, Figure 3A). Two of the brain regions that are relatively less affected by AD, and thereby represent best the normal biodynamic relationship between these cholinergic components. The protein levels of the synaptic AChE-S variant in CSF correlated with the baseline level of number of ^11^C-nicotinic binding sites in primary visual cortex (*r* = 0.81, *p* < 0.0002, Figure 3B), the pons (*r* = 0.60, *p* < 0.03), parietal cortex (*r* = 0.59, *p* < 0.033), cingulate gyrus (*r* = 0.56, *p* < 0.045), and the whole brain (*r* = 0.61, *p* < 0.019, Figure 3C).

The functional ChAT activity in CSF at baseline also correlated positively with the number of nicotinic binding sites in left thalamus (*r* = 0.60, *p* < 0.025) and right parieto-temporal cortex (*r* = 0.55, *p* < 0.03). We did not use the CSF Cholinergic index in these analyses since it is composed as a ratio between functional ChAT and AChE activities in CSF.

**Figure 4:**
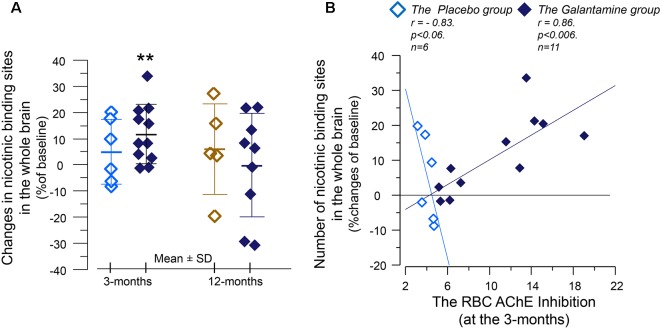
The relative changes of the *in vivo* nicotinic binding sites in the brain and association with changes in the red blood cells (RBCs) AChE activity. **(A)** Changes from baseline in the overall ^11^C-nicotine PET binding in the brain after 3 months of galantamine or placebo treatment (left panel) as well as after 9–12 months in all patients (right panel). **p < 0.007 compared to baseline. **(B)** Correlation between percent changes in the *in vivo* nicotinic binding sites and changes in the RBC AChE activity. The negative correlation among *the Placebo group* indicates that the co-dependency between the number of nicotinic binding sites and the AChE activity that was seen at baseline is still at work among *the Placebo group*. Note that AChE inhibition has inverse relationship with AChE activity. The galantamine treatment instead altered this basal relationship so that the nicotinic binding sites increase in accord with a reduction in (inhibition of) AChE activity by the drug. The *in vivo* nicotinic binding sites were assessed by PET using the ^11^C-nicotine tracer. Here is shown percent changes in the region of interest of the “Whole brain.” All values on both axes are percentages of the individual baseline. Light squares = *the Placebo* *group*. Dark squares = *the Galantamine group*. PET, positron emission tomography.

*At 3 months*, the number of nicotinic binding sites for the “whole brain regions” showed 11 ± 3% increases in *the Galantamine group* compared to baseline (*p* < 0.007, Figure 4A), which at the 12 months follow-up had returned to the levels at baseline (Figure 4A).

Analyses between the nicotinic binding sites and the RBC and CSF AChE activity showed positive correlation between the RBC AChE inhibition and the relative increases in the nicotinic binding sites in the majority of the brain regions, in particular in *the Galantamine group* (Figure 4B and Table 3). Noteworthy, the relative changes in RBC AChE activity among *the Placebo group* showed in contrast an inverse correlation with the number of nicotinic binding in the brain (Figure 4B). The latter most likely reflect a basal equilibrium condition between the access to ACh (regulated by AChE activity) and its nAChRs.

**Table 3:** Correlations between the changes in nicotinic binding sites in the brain assessed by PET and the RBC AChE inhibition after 3 and 12 months follow-up.

		The RBC AChE Inhibition					
		3-months				12-months	
		Placebo (*n* = 6)		Galantamine (*n* = 11)		Overall patients (*n* = 13)	
^11^C- nicotine binding in the brain regions (%1-*k*_2_*)		*r*	*p* < 0.05	*r*	*p* < 0.05	*r*	*p* < 0.05
Cortical regions	*L*	−0.79	0.07	0.61	**0.05**	−0.47	0.1
	*R*	−0.66	0.17	0.73	**0.009**	−0.51	0.07
Overall brain regions	*L*	−0.86	**0.03**	0.67	**0.02**	−0.53	0.07
	*R*	−0.66	0.17	0.70	**0.01**	−0.53	0.07
Average L & R cortical		−0.76	0.09	0.70	**0.05**	−0.50	0.08
Average L and R overall brain regions		−0.89	0.05*	0.71	**0.01**	0.54	0.06
Whole brain		−0.83	0.06*	0.76	**0.005**	−0.60	**0.03**

**Figure 5:**
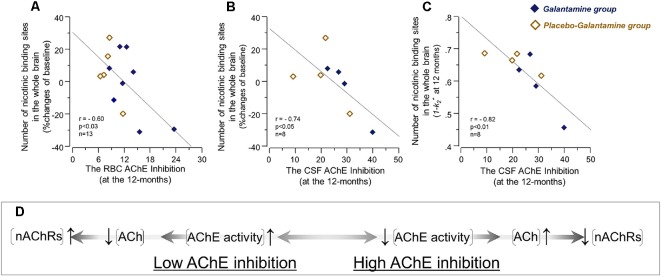
The initial co-dependency between the *in vivo* brain nicotinic binding sites and AChE activity was restored after 9–12 months treatment with galantamine. The graphs show negative correlations between the changes in the *in vivo* ^11^C-nicotine binding estimated by PET in the whole brain and the percent inhibition of RBC AChE activity **(A)**, and the percent inhibition of total AChE activity in CSF **(B)** following 9–12 months galantamine treatment. In panel **(C)** is shown the negative correlation between the absolute nicotinic binding (1-*k*_2_*) values with the percent AChE inhibition in CSF. In panel **(C)** the ^11^C-nicotine binding values are given as *1−k*_2_* since *k*_2_* values have an inverse relationship with this tracer’s binding, i.e., a low *k*_2_* values mean a higher ^11^C-nicotine binding level. The box **(D)** shows schematically a biodynamic model explaining the observed correlation between changes in the nicotinic binding and AChE activity at baseline (as shown in Figure 3) and here after 12 months of treatment (note that AChE inhibition shown here in graphs **(A–C)** have inverse relationship with AChE activity). In this model, high AChE inhibition (left-to-right on X-axis) means reduced AChE activity, which consequently means an increase in ACh level or a longer persistent of ACh in the synapses. This is balanced by adjustment of the nAChRs to lower levels (up-to-down on Y-axis). This might point at a protective feed-back mechanism against hyperexcitation state that may occur upon a too high AChE inhibition. Similarly, low AChE inhibition (right-to-left on X-axis) means higher AChE activity, which will reduce ACh levels and/or shorten its duration of action in the synapses. This is balanced by increases in the nAChR levels (down-to-up on Y-axis) to compensate for reduced level/duration of action of ACh. It should be noted that this model will not fit with the observations in *the Galantamine group* after 3 months treatment (as shown in Figure 4B), unless a general increase or remodeling of cholinergic-cholinoceptive interfaces is considered in the model, which would require simultaneous increases in both ChAT, AChE and nAChR levels, as was found in the current study. Open orange squares = *the Placebo-Galantamine* *group*. Dark squares = the *Galantamine group*. CSF, cerebrospinal fluid. PET, positron emission tomography; RBC, red blood cell; ACh, acetylcholine; AChE, acetylcholinesterase; nAChRs, nicotinic ACh receptors.

All r-values are correlation coefficient. A negative *r*-value reflects an inverse association between RBC AChE inhibition and the changes in the nicotinic binding sites in the brain. It should be noted that enzyme inhibition has an inverse relation with enzyme activity. Thereby, a negative *r*-value among *the Placebo group* means positive association between activity of the enzyme and the changes in the nicotinic binding sites in the brain. Overall, the negative *r*-value for this group in the table most likely point toward a native biodynamic relationship, meaning that a high ACh degradation (due to low or no inhibition of AChE) might become compensated by an increase in the number of nicotinic receptors to allow neurotransmission. This is because changes in RBC AChE activity or the nicotinic binding sites in the Placebo group during the initial 3 months of study are not caused by galantamine as these patients were on placebo. In contrast, the positive *r*-values found between these two variables in *the Galantamine group* indicate that a high AChE inhibition (i.e., a consequently high level and/or long duration of action of ACh within synapses) was accompanied by an increase in the number of nicotinic receptors in the brain. This in turn suggests that such cholinergic stimulation by the drug might induce neuronal and/or synaptic remodeling that requires more ACh receptors and cholinergic enzymes, such as ChAT and AChE to be expressed in the brain as we found in the current study (see also further schematic explanations in Figure 5D). Some of these correlations are graphically illustrated in Figure 4B. *k*_2_* values for ^11^C-nicotine binding are exemplified in Figure 3. *p-values are based on Spearman Rank Correlation; PET, Positron emission tomography, *k*_2_* = the binding constant. RBC AChE, Red blood cells acetylcholinesterase.

*At 3 months*, similar positive correlations were also observed among *the Galantamine group* between the relative changes in the nicotinic binding sites in the temporal medial lobe and the inhibitions of CSF AChE-S (*r* = 0.57, *p* < 0.04, *n* = 13) and AChE-R variants (*r* = 0.60, *p* < 0.03, *n* = 13).

No significant correlations were observed between ChAT_F_ activity or the Cholinergic index and the nicotinic binding sites in the brain in *the Galantamine* or *the Placebo* *group*.

*At 12 months*, the correlation analyses indicated an inverse association between the RBC AChE inhibition and the relative changes in the nicotinic binding sites in the brain regions (Figure 5A and Table 3). Similar inverse correlations were also observed between the inhibition of the CSF AChE and the relative 12-month changes in the nicotinic binding sites (Figure 5B and Table 4) as well as with absolute number of the nicotinic binding sites in the brain (Figure 5C).

**Table 4:** Correlations between the number of *in vivo* nicotinic binding sites and the inhibition of CSF AChE-S and AChE-R variants after 12 months.

		AChE-R variant (%Inhibition)		AChE-S variant (%Inhibition)		Total AChE (%Inhibition)	
The nicotinic binding sites in brain region (1-*k*_2_*)		*r* (*n* = 8)	*p* < 0.05	*r* (*n* = 8)	*p* < 0.05	*r* (*n* = 8)	*p* < 0.05
Cortical regions	*L*	−0.70	0.052	−0.67	0.059	−0.73	**0.037**
	*R*	−0.75	**0.028**	−0.70	**0.050**	−0.77	**0.022**
Overall brain regions	*L*	−0.76	**0.027**	−0.72	**0.043**	−0.78	**0.019**
	*R*	−0.79	**0.018**	−0.70	0.054	−0.79	**0.016**
Average cortical regions		−0.74	**0.034**	−0.71	**0.049**	−0.76	**0.025**
Average overall brain regions		−0.78	**0.019**	−0.72	**0.044**	−0.80	**0.015**
*Whole brain*		*−0.79*	***0.017***	*−0.75*	***0.029***	*−0.82*	***0.011***

All *r*-values are correlation coefficient. Some of the correlations are graphically illustrated in Figure 5. *k*_2_* = the binding constant for the PET tracer. The *k*_2_* values for ^11^C-nicotine binding are exemplified in Figure 3 and Figure 5C. PET, Positron emission tomography; CSF, Cerebrospinal fluid; AChE-S, Synaptic acetylcholinesterase splice variant protein; AChE-R, Read-through acetylcholinesterase splice variant. The percentages are calculated relative to the patients’ baseline value.

A mechanistic biodynamic model is presented in Figure 5D, which is based on the overall pattern of findings shown in Figures 3, 4B, 5A–C as well as Tables s 3, 4. Considering that low AChE inhibition (i.e., high AChE activity in synapses) means low concentration and/or short duration of action of ACh in the synaptic cleft (the left part of 5D), these findings suggest that the observed physiological equilibrium at the baseline condition between AChE activity and nAChRs (as shown in Figure 3 for all patients at baseline, and in Figure 4B and Table 3 for *the Placebo group*) was again restored following the longer-term of 9–12 months of galantamine treatment (as is shown in Figures 5A–C and Table 4).

*At 12 months*, no correlations were observed between the nicotinic binding sites in the brain and the CSF ChAT_F_ activity nor the CSF cholinergic index.

### Correlations Between Measures of Cognition and AChE and ChAT_F_ Activity in CSF

Descriptive data concerning changes in cognitive tests are reported in detail in the primary article (Kadir et al., 2008). Briefly, there was a statistically significant improvement in MMSE test at 3 months in *the Galantamine* but not *the Placebo* *group* compared to baseline. There was also a trend of improvement in ADAS-Cog test in *the Galantamine group* at 3 months (see Figures 5, 6 in Kadir et al., 2008). In the following, we hence focus on the correlations of these cognitive measures and the CSF cholinergic biomarkers.

Neither *at baseline* nor *at 3 months* there were any significant correlations between MMSE scores and the ChAT_F_ activity or the Cholinergic index in CSF.

**Figure 6:**
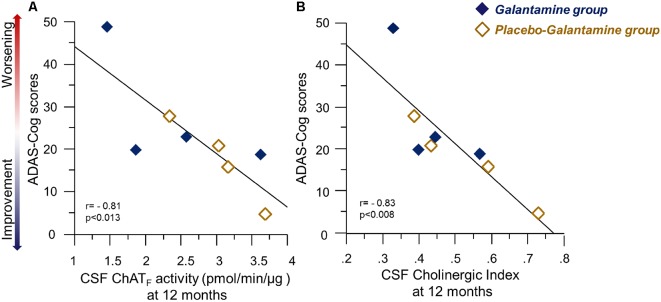
Correlations between ChAT_F_ activity or the Cholinergic index and the ADAS-Cog test results following 9–12 months of galantamine treatment. Panel **(A)** illustrates correlations between Cognition assessed by ADAS-Cog and the ChAT_F_ activity in CSF at 12 months. Panel **(B)** shows the corresponding correlation with Cholinergic index in CSF. It should be noted that a high ADAS-Cog score reflects a worse cognitive performance. Thus, the graphs illustrate positive correlation between the CSF cholinergic biomarkers and cognition. Open orange squares = the *Placebo-Galantamine* *group*. Dark squares = the *Galantamine group*. ChAT = choline acetyltransferase. CSF ChAT_F_ activity = the overall functional ChAT activity expressed in pmol/min/μg ChAT protein in the samples. CSF Cholinergic Index = ratio of ChAT_F_ activity to AChE_F_ in CSF.

At 12 months, there were significant correlations between several measures of cognition and the ChAT_F_ activity and Cholinergic index in CSF, which are summarized in Table 2. The negative correlations between ADAS-Cog and the CSF ChAT_F_ activity and the Cholinergic index are illustrated in Figure 6, which indicate those whose cognitive abilities worsened after 12 months of treatment had a lower ChAT_ F_ activity and Cholinergic index compared to the other patients.

## Discussion

Here, we described for the first time the effect of galantamine treatment on CSF levels of two important enzymes that maintain the steady state of ACh in the brain, in particular, the core-cholinergic enzyme responsible for biosynthesis of ACh, i.e., ChAT. The first 3 months of the study had a double-blinded placebo-controlled protocol, which allowed us to specifically address the biodynamic relationship of AChE and ChAT enzymes in response to galantamine treatment. We showed that specific inhibition of the CSF AChE activity by galantamine treatment was accompanied by a mild increase of the activity of ChAT_F_ in CSF at 3 months follow-up, with no changes seen in *the Placebo group* regarding the activities of these two enzymes. Furthermore, we found that upon switching from placebo to active treatment a similar pattern of changes in CSF ChAT_F_ and AChE activities appeared when measured at the 12-months follow-up. We therefore defined a new CSF cholinergic biomeasure by integrating the activities of functional ChAT and AChE in the CSF as a ratio, i.e., the CSF Cholinergic index, for monitoring changes in ACh homeostasis in the CSF (and the brain) following treatment with ChEIs, like galantamine. This Cholinergic index showed a clear increase as response to ChEI-treatment.

In this study, we further assessed the changes in these CSF parameters with the clinical and paraclinical measures in the patients. This way we showed presence of a dynamic pattern of interdependency of the CSF ChAT_F_ activity, the Cholinergic index and the AChE activity to the *in vivo* AChE inhibition and nicotinic binding sites in several brain regions assessed by PET in AD patients prior to and after treatment with galantamine.

Previously, we have shown that galantamine treatment causes 30%–36% of *in vivo* AChE inhibition in the majority of the regions of the brain, in particular the cortical regions in these patients assessed by PET (Kadir et al., 2008). Here, we found that this *in vivo* AChE inhibition correlated with both CSF ChAT_F_ activity and the cholinergic index. This is the first study showing that *in vivo* AChE inhibition induces an increase in ChAT_F_ activity in the CSF of patients following treatment with a ChEI. These findings may indicate that galantamine indirectly affect the activity of the cholinergic neurons as well as the down-stream cholinoceptive neurons. In other words, ChEIs therapy may both prevent degradation of ACh as well as by increasing ChAT_F_ levels increase the production of ACh.

We further found that galantamine treatment was associated with a 25%–30% inhibition of the total AChE activity in CSF compared to pre-treatment levels and placebo treatment. This is consistent with our previous report showing similar levels of inhibition of the synaptic AChE-S variant in CSF of the galantamine-treated patients (Darreh-Shori et al., 2008).

We then looked at the interdependency of these changes with *in vivo* nicotinic binding sites in the brain assessed by ^11^C-nicotine PET. Although we found no correlation between the CSF ChAT_F_ activity and the *in vivo* nicotinic binding sites, significant correlations were observed between the *in vivo* nicotinic binding sites and the RBC and CSF AChE activities in the AD patients. At the baseline condition, we found a positive association between the AChE-S activity and the *in vivo* brain nicotinic binding sites. This was particularly prominent in the primary visual cortex and pons, which are two brain regions known to be relatively less affected by AD. Thus, these findings imply that a high AChE activity is physiologically counterbalanced by high *in vivo* nicotinic binding sites in the brain of AD patients (as depicted schematically in Figure 5D), possibly due to low availability and/or short duration of action of ACh. This is consistent with the observations in the AChE over-expressing transgenic mice, in which the high AChE activity seemed to be compensated by an elevation of the number of cortical nAChRs (Svedberg et al., 2002, 2003; Mousavi et al., 2004). These observations hence indicate the presence of a basal physiological equilibrium between ACh and its target receptors, i.e., here the nAChRs. In other words, these findings suggest that under baseline conditions, AD patients with high AChE levels also have a high number of nAChRs in the brain, most likely reflecting a physiological equilibrium (or compensation) between available concentration and/or duration of action of ACh and levels of AChE-S activity in the synaptic clefts.

A comparison at the 3-months follow-up between *the Placebo* and *the Galantamine groups* indicated that the baseline positive association between the AChE activity and the nicotinic binding sites was not altered among *the Placebo group*, while in *the Galantamine-treated group* a reduction in AChE activity (i.e., AChE inhibition) was instead accompanied by an increase in the *in vivo* nicotinic binding sites in the majority of the brain regions. Intuitively, these findings appear unexpected and paradoxical with regards to the pattern of the observations at the baseline. This is because a high AChE inhibition implies less ACh degradation and thereby a prolonged action of ACh on its receptors within the synaptic cleft (as depicted in Figure 5D). This in physiological terms should lead to a reduction and not an increase in the numbers of nAChRs (as shown in Figure 4B). Thus, the observed mild 11% increase in the *in vivo* nicotinic binding sites in the brain of *the Galantamine-treated* patients can be explained, in a mechanistic view, by an increase in synaptic regeneration and/or synaptic strengthening at the neuronal interfaces between cholinergic and cholinoceptive neurons. This is also in line with the observed increased levels of both ChAT_F_ and the Cholinergic index in the CSF of these patients in response to a higher *in vivo* brain AChE inhibition. Indeed, the rationale for the use of ChEIs is to stimulate the cholinergic neuronal network. Thus, galantamine-induced AChE inhibition is expected to lead to a more proper cholinergic signaling. This, in turn, might rescue the under-stimulated cholinergic-cholinoceptives neuronal interfaces and/or induce synaptic remodeling and regeneration in the brain. The required time for such re-modulation also coincides with the top level of clinical manifestation of cognitive improvement that is seen within 3–6 months of therapy with ChEIs (Raskind et al., 2000; Tariot et al., 2000). The correlations between RBC AChE activity and the ^11^C-nicotine PET also suggest that measurement of RBC AChE activity might be of value as a general index for the overall nicotinic receptors in the brain. Indeed, a study has shown that RBC AChE measurement might have the potential as a peripheral biomarker for diagnosis of familial Alzheimer’s and Parkinson’s disease dementia (Bawaskar et al., 2015).

*At 12 months* follow-up, i.e., when all patients were on 9–12 months treatment, the baseline equilibrium seemed to be reestablished, in other words, the positive correlation between the *in vivo* nicotinic binding sites and the AChE activity. Experimental animal studies are also in line with our findings and the described time-dependent biodynamic model of neuronal synaptic plasticity and/or remodeling of the cholinergic and the related neuronal networks. In non-transgenic mice, a similar increase in nAChRs was observed after short-term galantamine treatment in hippocampus, thalamus and several cortical regions compared to saline-treated mice (Svedberg et al., 2004). In contrast, no significant changes were observed in the AChE overexpressing littermate after galantamine treatment (Svedberg et al., 2004). In *APPsw* transgenic mice, galantamine treatment leads to an increase in the protein level of synaptophysin in the cortex compared to saline-treated control (Unger et al., 2006). Synaptophysin together with synaptogyrin seems to play essential roles in synaptic plasticity (Janz et al., 1999). Interestingly, the regulation of synapse formation by synaptophysin appears to be activity-dependent (Tarsa and Goda, 2002).

In addition, both CSF ChAT_F_ and the Cholinergic index at 12 months showed strong positive correlations with the *in vivo* AChE activity supporting the notion that a new balance between the degradation and production of ACh was established in the brain. Indeed, we found that both CSF ChAT_F_ and the Cholinergic index also correlated with several cognitive measures at this follow-up time. Similar correlations with MMSE and ADAS-cog have been reported following treatment with encapsulated nerve growth factor releasing implants in nucleus basalis of Meynert of AD patients (Karami et al., 2015), which is another cholinergic enhancing therapeutic strategy. Thus, the CSF ChAT_F_ and the Cholinergic index may be useful as a monitoring biomeasure following cholinergic enhancing treatments.

In this context, the important questions are how and when treatment with ChEIs (or even with a fully disease-modifying drug) may be able to be manifested as improvement in the CSF cholinergic index, or simply how this index may differ among patients at different stage of the disease. Here, it is crucial to refer to the rational for treatment with ChEIs. These drugs by definition require a certain amount of ACh production (by ChAT) and release into the synapses. In other words, these drugs will not work if the production and release of ACh into synapses is heavily affected or is absent. Given that the cholinergic neurons degenerate early and progressively in the course of AD, it is expected that each remaining cholinergic neuron will with time have fewer synaptic interfaces at its projection sites in the brain that fulfill this criteria as the disease progresses. Thereby, it is plausible that not all of the degenerating synapses of a cholinergic neuron can be stimulated by treatment with ChEIs. This might explain the limited and moderate clinical efficacy observed following treatment with ChEIs. Given that even at mild stage of AD, a substantial number of cholinergic synapses may have already degenerated beyond the rescue point, it is not very surprising that the observed increase in soluble ChAT activity was quite mild in the current study following galantamine treatment. This, in turn, suggests that a higher degree of increase in soluble ChAT should be expected if treatment initiated at milder stage of the disease or even at asymptomatic stages.

This study has several limitations. It was primarily designed as a PET investigation, thus only included a small group of mild AD patients. These features make it necessary to be cautious when interpreting the data, since the small sample size may result in a lack of statistical power, although this might make the significant results even more interesting. Also, relatively large numbers of statistical analyses were performed on the PET measurement in relation to the CSF cholinergic biomarkers and cognitive function, which were not corrected for multiple comparisons. This may render some of the results liable to be obtained by chance. Therefore, we weighted the overall patterns of the results rather than isolated findings. The number of available data at the 12 months follow-up was particularly small because either no CSF sample or PET scan was available for a few of the patients.

In summary, we report for the first time that a cholinesterase inhibitor increases the CSF level of the ACh synthesizing enzyme, choline acetyltransferase. We also report here distinct time and treatment-dependent associations between the CSF ChAT_F_ activity, the RBC or CSF AChE activity (or inhibition) and the *in vivo* AChE activity and nicotinic binding sites in the brain, measured by PET. The pattern of the findings indicates a time-dependent biodynamic remodeling and/or reorganization, indicative of establishment of a new bio-equilibrium in the function of the cholinergic neuronal interfaces following ChEI-treatment vs. placebo as well as the treatment duration. Altogether, these observations suggest that a neuronal and/or synaptic remodeling occurred in response to stimulation with a ChEI’s treatment, which in turn provide a mechanistic basis for the transient stabilization of cognition and delayed progression of the disease observed in AD patients following therapy with ChEIs (Giacobini, 2000, 2001).

## Data Availability Statement

The datasets generated for this study are available on request to the corresponding author.

## Ethics Statement

The studies involving human participants were reviewed and approved by The Ethics Committee of Huddinge University Hospital, Sweden. The patients/participants provided their written informed consent to participate in this study.

## Author Contributions

TD-S and AKarami conceived and designed the CSF experiments; set up and performed the CSF AChE and ChAT assays and performed statistical analysis of the data. AKarami, TD-S and ME wrote the first draft and did the initial revisions. AN conceived and designed the original galantamine-PET study. AKadir and AN did the *in vivo* AChE and nicotine binding analyses. OA performed the neuropsychological assessments on the patients. All authors read and approved this final draft of the manuscript.

## Conflict of Interest

The authors declare that the research was conducted in the absence of any commercial or financial relationships that could be construed as a potential conflict of interest.

## Supplementary Material

FIGURE S1 Changes in the ChAT_F_ based on patients whose CSF were available both at 3 and 12 months treatment for (RM) ANOVA analysis. The graphs **(A,B)** are a modified version of the corresponding graphs 1C and 1D in Figure 1. Here, data from one subject in the placebo (3 months follow-up) is omitted since no CSF at 12 months was left for these patients. Thereby paired analyses could be done on four subjects. This sub-analyses in **(A)** shows that the *Galantamine group* had about 50% higher levels of ChAT_F_ in CSF than *the Placebo group* at 3 months, (here *n* = 4, *p* < 0.05). At 12 months *the Placebo-Galantamine group* exhibited about 30% significant increase in the CSF ChAT_F_ activity compared to their own 3 months follow-up (*n* = 4, *p* < 0.02). Panel **(B)** shows similar sub-analyses with regards to changes in the cholinergic index. At 3 months, the actual differences between the *Galantamine group* and *the Placebo group* (*n* = 4) was over 70%. At 12 months, the cholinergic index was increased over 50% in *the Placebo-Galantamine group* compared to their own 3 months follow-up (*n* = 4, *p* < 0.02). **p* < 0.05 signify differences compared to *the Placebo group* at 3 months. ^#^*p* < 0.02 signify differences compared to *the Placebo group* at 3 months. CSF ChAT_F_ activity = the overall functional ChAT activity expressed in pmol/min/μg ChAT protein in the samples.
