# Supplementary figures and images for: CSF Cholinergic Index, a New Biomeasure of Treatment Effect in Patients With Alzheimer’s Disease
Source: Front Mol Neurosci. 2019 Oct 11;12:239. doi: 10.3389/fnmol.2019.00239 (PMC6798072; doi:10.3389/fnmol.2019.00239)

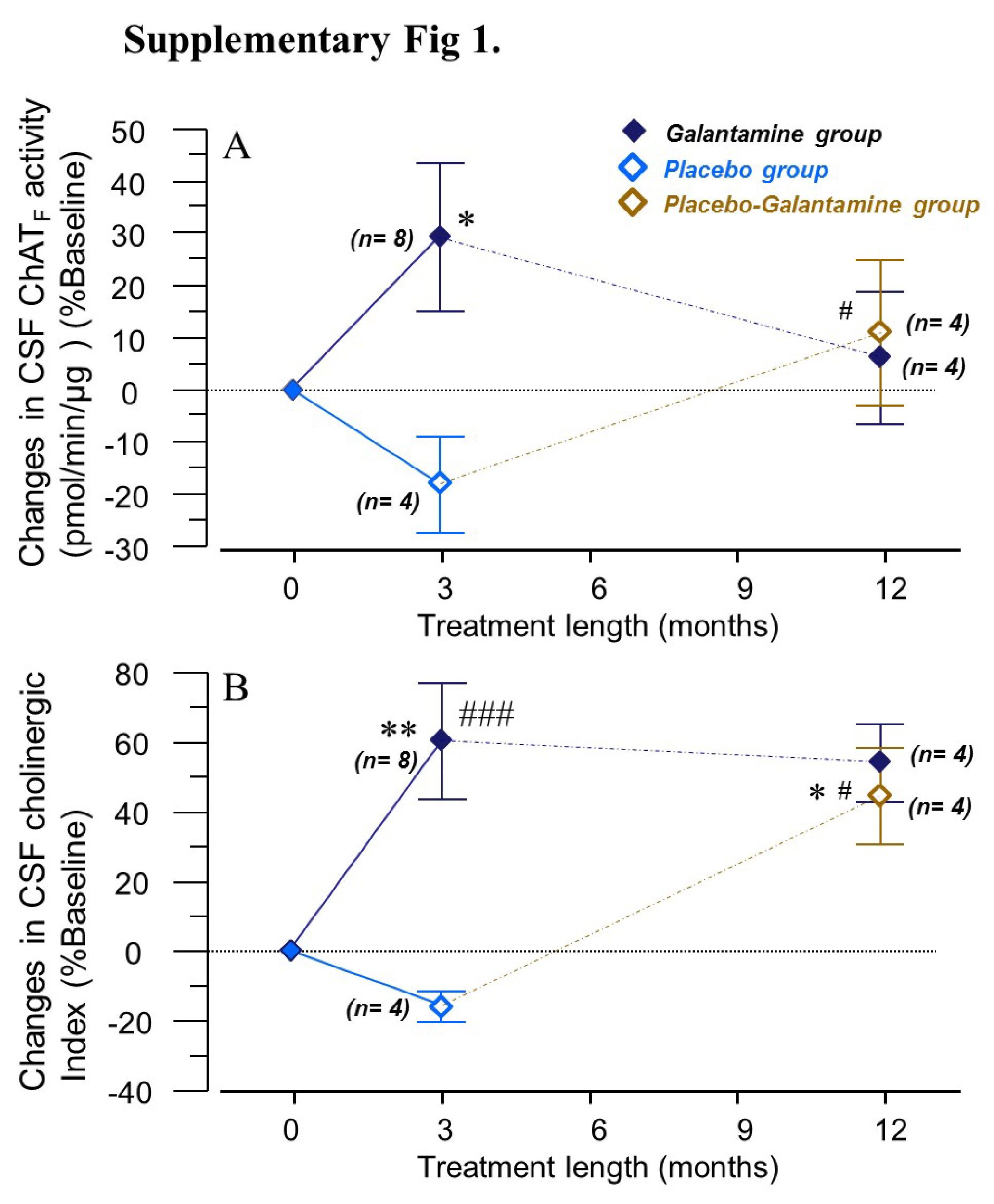

Supplement: FIGURE S1 — Changes in the ChATF based on patients whose CSF were available both at 3 and 12 months treatment for (RM) ANOVA analysis. The graphs (A,B) are a modified version of the corresponding graphs 1C and 1D in Figure 1. Here, data from one subject in the placebo (3 months follow-up) is omitted since no CSF at 12 months was left for these patients. Thereby paired analyses could be done on four subjects. This sub-analyses in (A) shows that the Galantamine group had about 50% higher levels of ChATF in CSF than the Placebo group at 3 months, (here n = 4, p < 0.05). At 12 months the Placebo-Galantamine group exhibited about 30% significant increase in the CSF ChATF activity compared to their own 3 months follow-up (n = 4, p < 0.02). Panel (B) shows similar sub-analyses with regards to changes in the cholinergic index. At 3 months, the actual differences between the Galantamine group and the Placebo group (n = 4) was over 70%. At 12 months, the cholinergic index was increased over 50% in the Placebo-Galantamine group compared to their own 3 months follow-up (n = 4, p < 0.02). *p < 0.05 signify differences compared to the Placebo group at 3 months. #p < 0.02 signify differences compared to the Placebo group at 3 months. CSF ChATF activity = the overall functional ChAT activity expressed in pmol/min/μg ChAT protein in the samples. [file Image_1.JPEG]
